# The effectiveness of the Austrian disease management programme for type 2 diabetes: a cluster-randomised controlled trial

**DOI:** 10.1186/1471-2296-11-86

**Published:** 2010-11-05

**Authors:** Andreas C Sönnichsen, Henrike Winkler, Maria Flamm, Sigrid Panisch, Peter Kowatsch, Gert Klima, Bernhard Fürthauer, Raimund Weitgasser

**Affiliations:** 1 Institute of General Practice, Family Medicine and Preventive Medicine, Paracelsus Medical University, Strubergasse 21, 5020 Salzburg, Austria; 2 Workinggroup for Preventive Medicine, Salzburg (AVOS), Elisabethstr. 2, 5020 Salzburg, Austria; 3 Styrian Public Health Insurance, Josef-Pongratz-Platz 1, 8011 Graz, Austria; 4 Austrian Association of General Practice (ÖGAM), Wiener Medizinische Akademie, Alser Str. 4, 1090 Wien, Austria; 5 Medical Department, Diakonissen-Hospital, Guggenbichlerstr. 20, 5026 Salzburg, Austria

## Abstract

**Background:**

Disease management programmes (DMPs) are costly and impose additional work load on general practitioners (GPs). Data on their effectiveness are inconclusive. We therefore conducted a cluster-randomised controlled trial to evaluate the effectiveness of the Austrian DMP for diabetes mellitus type 2 on HbA1c and quality of care for adult patients in primary care.

**Methods:**

All GPs of Salzburg-province were invited to participate. After cluster-randomisation by district, all patients with diabetes type 2 were recruited consecutively from 7-11/2007. The DMP, consisting mainly of physician and patient education, standardised documentation and agreement on therapeutic goals, was implemented in the intervention group while the control group received usual care. We aimed to show superiority of the intervention regarding metabolic control and process quality. The primary outcome measure was a change in HbA1c after one year. Secondary outcomes were days in the hospital, blood pressure, lipids, body mass index (BMI), enrolment in patient education and regular guideline-adherent examination. Blinding was not possible.

**Results:**

92 physicians recruited 1489 patients (649 intervention, 840 control). After 401 ± 47 days, 590 intervention-patients and 754 controls had complete data. In the intention to treat analysis (ITT) of all 1489 patients, HbA1c decreased 0.41% in the intervention group and 0.28% in controls. The difference of -0.13% (95% CI -0.24; -0.02) was significant at p = 0.026. Significance was lost in mixed models adjusted for baseline value and cluster-effects (adjusted mean difference -0.03 (95% CI -0.15; 0.09, p = 0.607). Of the secondary outcome measures, BMI and cholesterol were significantly reduced in the intervention group compared to controls in ITT after adjustments (-0.53 kg/m²; 95% CI -1.03;-0.02; p = 0.014 and -0.10 mmol/l; 95% CI -0.21; -0.003; p = 0.043). Additionally, more patients received patient education (49.5% vs. 20.1%, p < 0.0001), eye- (71.0% vs. 51.2%, p < 0.0001), foot examinations (73.8% vs. 45.1%, p < 0.0001), and regular HbA1c checks (44.1% vs. 36.0%, p < 0.01) in the intervention group.

**Conclusion:**

The Austrian DMP implemented by statutory health insurance improves process quality and enhances weight reduction, but does not significantly improve metabolic control for patients with type 2 diabetes mellitus. Whether the small benefit seen in secondary outcome measures leads to better patient outcomes, remains unclear.

**Trial Registration:**

Current Controlled trials Ltd., ISRCTN27414162.

## Background

The prevalence of type 2 diabetes is rising worldwide for all age groups due to population growth, ageing, urbanisation, increasing prevalence of obesity and physical inactivity [1,2]. In Austria at least 300,000- 315,000 patients have diabetes type 2 (4.2 - 4.6% of the adult population) [3]. The prevalence of late diabetic complications corresponds to the European average as depicted in the CODE-2-study [4]. In Austria, deficits in implementation of standard care for type 2 diabetes exist, and there appears to be a strong demand for management optimisation [5].

The chronic care model (CCM) has been developed to improve the care for patients with chronic conditions like diabetes mellitus type 2 [6]. Disease management programmes (DMPs) consisting of physician training in guideline-adherent therapy, patient education, patient and physician reminders and continuous feedback have been introduced to implement the CCM in practice. While it has been shown that interventions containing at least one component of the CCM are effective in improving care [7], the benefits of DMPs are still discussed controversially. In Germany, the nation-wide mandatory implementation of DMPs by statutory public health insurances may have led to increasing bureaucracy rather than to an improvement in care and therefore the programmes have been criticised widely [8]. Current evaluation studies in Germany appear to reveal benefits of the DMP regarding mortality, but are of limited validity due to selection bias and retrospective methodology [9]. A former evaluation study in Upper Austria showed positive results regarding the effectiveness of disease management. This programme, too, has not been evaluated in a randomised controlled trial, and has been restricted to small samples of highly motivated physicians [10]. Thus, the current evidence base remains insufficient to support a general implementation of DMPs. Large programmes of statutory public health insurances have never been evaluated in randomised controlled studies. Published data of randomised controlled trials of private health insurers and in community health settings show only limited and inconsistent success regarding surrogate measures. To date, only one randomised controlled trial investigating the outcome of a DMP programme has been published [11]. The study demonstrated improvement in glycemic control but had no impact on cardiovascular morbidity and mortality after six years of observation.

In summary, published data on the effectiveness of DMPs for diabetes mellitus type 2 are inconsistent and inconclusive. These findings show the necessity of thorough evaluation of newly designed DMPs by a randomised controlled trial before general implementation. This is especially true for large public programmes that impose additional work on the surgeries involved and additional costs to the health care system. These costs may only be justified, if the effectiveness of the programme is proven.

As public health interventions tend to be complex and context dependent, evaluation of effectiveness must be sufficiently comprehensive to account for this complexity. Randomised controlled trials have been described as the best method for appraising a causal relationship between a complex intervention and clinical outcomes [12]. In several instances such as the implementation process of DMPs by public health insurances in Germany, the chance for a rigorous evaluation was missed due to prior general implementation, leading to persistent scepticism regarding the effectiveness of these programmes.

Nationwide implementation of a DMP for type 2 diabetes called "Therapie aktiv" is currently underway in Austria. We evaluated whether this DMP designed by the Austrian statutory public health insurance leads to an improvement of metabolic control (HbA1c) and process quality of care in adults with diabetes mellitus type 2 compared to controls. This is the first study to evaluate a DMP implemented by statutory public health insurance using a randomised controlled design.

## Methods

### Design

The study was performed as a pragmatic cluster-randomised controlled superiority trial of a complex intervention with an observation time of one year. Randomization was carried out at the district level of the Salzburg province which resulted in a 3-level cluster design in which the surgery was nested within the district, and patients were nested within the surgeries. Randomisation at the patient level would have lead to contamination effects because a single GP could not treat certain patients according to usual care and others according to the DMP. Randomisation at the GP level would have led to contamination effects because of overlapping patient groups, especially in rural areas.

The study took place in the province of Salzburg with a total population of about 500,000 where the estimated prevalence of type 2 diabetes (about 2.5 to 3%) is lower than the Austrian average [3].

### Participants

Participation in the study was offered to all 275 primary care physicians having a contract with the public health insurance (252 GPs and 23 internists providing primary care). We informed all physicians both in writing and by telephone about the study and its objectives and asked them to declare their willingness to participate by letter or fax. 98 physicians signed up, 88 GPs and 10 internists involved in primary care.

Participating physicians were encouraged to continuously recruit all patients with diabetes type 2 who entered the surgery during the recruitment period (from July 15 to November 30 of 2007). To avoid differential recruitment, all patients were asked to declare their willingness to participate in the DMP. Control patients were told that they would enrol in the DMP after one year. All patients willing to participate were included in the study after informed consent according to the Declaration of Helsinki. Exclusion criteria were dementia/psychiatric illness with inability to participate or to give informed consent. Patients suffering from a disease with limited life expectancy such as advanced cancer were not included as these patients were unlikely to participate in the DMP after standard implementation.

### Intervention

The DMP "Therapie aktiv" intervention included the following components:

- A mandatory 10-hour face to face training course for physicians, designed by the Austrian Diabetes Association (ÖDG), the Austrian Medical College (Ärztekammer), and the Austrian Society for General Practice (ÖGAM) consisting of an update in diabetes care, current guidelines of the ÖDG, and practice management training.

- Nine hours of patient-education in 4 modules with a group size of 3 to 12 patients. Patient education was organised by the Working Group for Preventive Medicine Salzburg (AVOS) using the „Düsseldorfer Modell" curriculum [13,14]. Training was conducted by physicians in their surgeries or in out-patient clinics. Prior to the implementation of the DMP, these patient modules were offered throughout the province but not used widely.

- Standardised documentation of physical examination, laboratory findings, and diabetes complications in a DMP-form once a year.

- Structured interdisciplinary care according to the guidelines of the Austrian Diabetes Association (ÖDG) [15].

- Agreement on therapeutic goals in a shared patient-physician decision-making process at three-monthly intervals.

In the control group, physicians performed usual care. The physicians of the control group were not permitted to participate in the 10-hour DMP training course. As patient education for diabetes has been publicly available, participation was possible for controls on a voluntary basis, but patients were not explicitly invited to participate.

### Baseline examination

At inclusion the following measures were examined: HbA1c, cholesterol, triglycerides, LDL-and HDL-cholesterol, height and body weight, systolic and diastolic blood pressure. Laboratory tests were performed in a central laboratory contracted by public health insurance. The laboratory-staff were blinded as to whether the samples came from the intervention group or from controls. Anthropometric measurements were taken by the participating physician. Blinding of the physicians was not possible.

### Final examination

After one year the baseline examination was repeated. In an additional case report form, diagnostic measures (i.e. HbA1c-checks, ophthalmological and foot examinations) and participation in patient education were recorded.

### Objectives of the study

The study focuses on the question whether the Austrian DMP "Therapie aktiv" designed and implemented by statutory public health insurance improves metabolic control (HbA1c) and quality of care for adults with type 2 diabetes managed in primary care compared to a control group with usual diabetes care. We hypothesised that the DMP would lead to a significant reduction of HbA1c and an improvement in guideline adherent care (process quality).

### Outcome measures

The primary endpoint of the trial was determined to be the change in HbA1c from baseline to 12 months (final examination). Secondary outcomes included an improvement in systolic or diastolic blood pressure, lipids, and body mass index. Furthermore, we analysed measures of process quality including the frequency of HbA1c measurements, eye and foot examinations as well as participation in patient education.

### Sample size

Sample size was calculated using the expected change in HbA1c from baseline to the final examination. Using an estimate of standard deviation for HbA1c change of 2%, a total of 504 patients (252 per arm) was required to detect a difference of HbA1c of 0.5% with a power of 1-β = 80% using a two-sided two-sample T-Test at a 0.05 significance level. The estimated intra-cluster correlation coefficient was 0.05 at the level of the surgery and negligible at the district level. In order to have adequate power, the sample size had to be increased to 984 patients (492 per arm). Assuming a drop-out-rate of 20%, the sample size was adjusted to 615 patients per arm, or a total of 1230 patients.

### Randomisation

To assure concealment of allocation at the physician level, GPs and internists were not told whether they would be in the intervention or the control group until after obtaining their consent to participate. After completion of physician recruitment, cluster-randomisation at the level of the districts was performed with computerised sequence generation. To assure even distribution of the districts regarding population characteristics (urban, rural, mixed) and size, the districts were randomised as matched pairs. As the only urban region of the province, the city of Salzburg was divided into two study districts, and the small mountain-districts (Pinzgau and Lungau) were combined into one study district yielding a total of 6 study districts: two urban districts (Salzburg city left to the Salzach river, Salzburg city right to the Salzach river), two rural districts (Pinzgau-Lungau and Pongau), and two mixed districts (Tennengau and Flachgau). Thus randomisation led to both intervention and control groups containing one urban, one rural and one mixed district. Blinding of physicians or patients was not possible due to the complexity of the intervention.

### Statistical methods

All data were recorded in the surgeries of the participating physicians and transferred to the Institute of General Practice, Family Medicine and Preventive Medicine of the PMU for further processing and evaluation. We analysed differences between groups using mixed models in IBM^® ^SPSS^® ^Statistics18.0 to adjust for baseline characteristics and cluster effects. The Fisher's Exact Test was used to check for significant differences regarding parameters of process quality (i.e. percentage of patients with guideline adherent care). Within-group-differences in pre-post-analysis were tested using the T-Test for paired samples.

### Ethical issues and trial registration

This trial has been approved by the ethics committee of Salzburg, Austria, and has been registered with Current Controlled Trials Ltd. (ISRCTN27414162) on July 12, 2007.

Further details of the methodology and study protocol have been published elsewhere [16].

## Results

**Figure 1:**
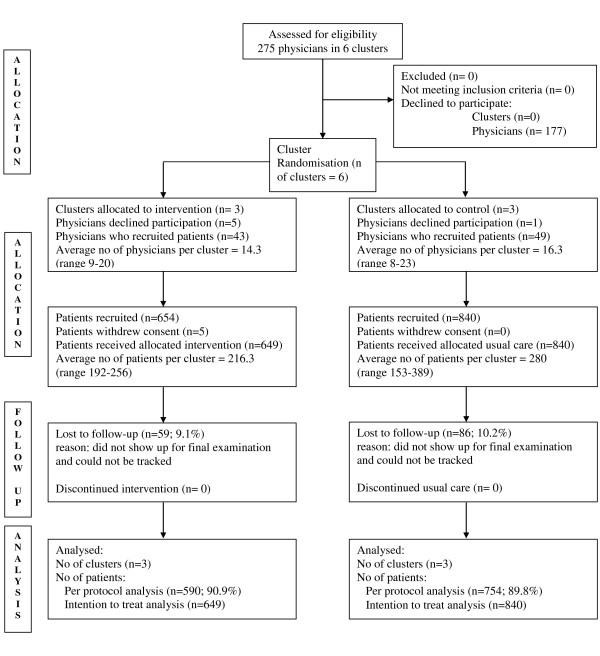
**Flow of Clusters and Participants**.

98 of 275 (35.6%) physicians eligible signed up to participate. Five physicians of the intervention group and one physician of the control group dropped out before recruiting patients. The remaining 92 physicians (43 intervention group, 49 control group) recruited 1494 patients, 654 in the intervention and 840 in the control group. In the intervention group, five patients were excluded because they withdrew consent prior to the documentation of baseline data. 9.1% (n = 59) of the patients in the intervention group and 10.2% (n = 86) of the patients in the control group were lost to follow up and could not be tracked due to moving away or non-compliance. Details of the flow of clusters, physicians and patients through the study are shown in figure 1 as suggested by the consort statement for cluster randomised trials [17].

Because of the significantly higher number of patients in the control group, an analysis was carried out to exclude differential recruitment and selection bias. Based on the larger population size in the control districts, more physicians were included in the control group. The population size per eligible physician was comparable (5773 persons/physician in the intervention group and 5670 persons/physician in the control group). Neither the participation rate of physicians nor the number of patients recruited per physician differed significantly between groups (table 1).

**Table 1:** Numbers of participating physicians and patients

	Intervention	Control	p-value
Medical doctors eligible	125	150	

Medical doctors in the study (n)	43	49	

Medical doctors in the study (% of eligible MDs)	34.4	32.7	0.80^1^

Patients recruited	649	840	

Patients recruited/medical doctor	15.1	17.1	0.45^2^

^1 ^Fisher's Exact Test

^2 ^Mann-Whitney-Test

Baseline data are shown in table 2. There were no significant differences between the intervention and the control group except for BMI and cholesterol, with intervention patients being slightly heavier and having higher cholesterol levels than controls.

**Table 2:** Baseline data

	Intervention	Control	p-value
Number of patients at baseline	649	840	

Percentage of women	49.0%	46.9%	0.43^1^

Age (years ± SD)	65.4 ± 10.4	65.5 ± 10.4	0.95^2^

HbA1c (% ± SD)	7.46 ± 1.53	7.34 ± 1.31	0.10^2^

Creatinine (μmol/l ± SD)	84.86 ± 30.94	84.86 ± 34.48	0.92^2^

Triglycerides (mmol/l ± SD)	2.14 ± 1.82	2.00 ± 1.73	0.12^2^

Cholesterol (mmol/l ± SD)	5.15 ± 1.14	5.02 ± 1.09	0.02^2^

HDL (mmol/l ± SD)	1.35 ± 0.39	1.32 ± 0.36	0.60^2^

LDL (mmol/l ± SD)	2.87 ± 0.96	2.87 ± 0.91	0.78^2^

Systolic blood pressure (mmHg ± SD)	141 ± 19	139 ± 17	0.12^2^

Diastolic blood pressure (mmHg ± SD)	83 ± 11	82 ± 10	0.41^2^

BMI (kg/m² ± SD)	30.4 ± 5.1	29.7 ± 4.9	0.01^2^

^1 ^Fisher's Exact Test

^2 ^independent T-Test

After an average of 401 ± 47 days (range 300-647), 90.9% of the intervention group (590 patients) and 89.8% of the control group (754 patients) had complete data regarding primary outcome measure. The number of patients with complete data for secondary outcome measures varied slightly (see table 3). Because of the realities of adherence and retention in a programme such as the DMP, we evaluated our data in an intention-to-treat (ITT) approach using the last available data carried forward method. With regards to primary outcome measure, we found a decrease in the HbA1c of 0.41% (95% CI 0.32; 0.50) in the intervention group and a decrease of 0.28% (95% CI 0.21; 0.35) in the control group. The pre-post-comparison was significant at a level of p < 0.0001 for both groups (table 3). Secondary outcome measures including triglycerides, BMI, systolic and diastolic blood pressure also decreased significantly in the intervention group in pre-post analysis, but not in the control group (table 3).

**Table 3:** Changes within groups in primary and secondary outcome measures^0^

Reduction of	Intervention				Control			
	**n^1^**	**mean^2^**	**95% CI**	**p-value^3^**	**n^1^**	**mean**	**95% CI**	**p-value^3^**

primary outcome measure								

HbA1c (%)	590	0.41	[0.32; 0.50]	<0.0001	754	0.28	[0.21; 0.35]	<0.0001

secondary outcome measures								

Creatinine (μmol/l)	586	-0.88	[-2.65; 0.88]	0.165	739	0.88	[-0.88; 2.65]	0.504

Triglycerides (mmol/l)	585	0.15	[0.02; 0.27]	0.021	736	0.09	[0.00; 0.17]	0.046

Cholesterol (mmol/l)	585	0.06	[-0.01; 0.14]	0.112	736	0.01	[-0.06; 0.07]	0.797

HDL (mmol/l)	585	-0.02	[-0.04; -0.01]	0.011	736	-0.01	[-0.03; 0.01]	0.209

LDL (mmol/l)	585	-0.01	[-0.08; 0.07]	0.864	736	0.04	[-0.02; 0.10]	0.161

RR systolic (mmHg)	561	2.49	[1.03; 3.94]	0.001	691	0.68	[-0.46; 1.83]	0.241

RR diastolic (mmHg)	561	1.16	[0.35; 1.98]	0.005	691	0.61	[-0.08; 1.30]	0.084

BMI	568	0.31	[0.17; 0.44]	<0.001	695	0.04	[-0.09; 0.16]	0.540

^0 ^Reduction is shown as positive number.

^1 ^n per protocol

^2 ^intention to treat

^3 ^paired T-Test

Our ITT analysis using unadjusted between-group-analysis demonstrated significant reduction of HbA1c (-0.13%; 95% CI -0.24; -0.02) and BMI (-0.27 kg/m²; 95% CI -0.45; -0.08) (p = 0.026 and 0.004 respectively, table 4). We calculated intra-cluster correlation coefficients (ICC) for both levels of clustering (table 5) [18-20], and then used mixed models to adjust for cluster effects and baseline value. After adjustment, only weight loss and cholesterol-reduction were significantly larger in the intervention group than in controls (p = 0.040 and 0.043 respectively, table 5).

**Table 4:** Differences between groups regarding primary and secondary outcome measures

	mean difference^1^	95%-CI	p-value^2^
Primary outcome measure			

HbA1c (%)	-0.13	[-0.24; -0.02]	0.026

Secondary outcome measures			

Creatinine (μmol/l)	1.77	[-0.88; 4.42]	0.157

Triglycerides (mmol/l)	-0.06	[-0.21; 0.09]	0.417

Cholesterol (mmol/l)	-0.05	[-0.16; 0.05]	0.291

HDL (mmol/l)	0.01	[-0.01; 0.04]	0.295

LDL (mmol/l)	0.05	[-0.04; 0.14]	0.314

RR systolic (mmHg)	-1.80	[-3.65; 0.05]	0.057

RR diastolic (mmHg)	-0.55	[-1.62; 0.51]	0.307

BMI	-0.27	[-0.45; -0.08]	0.004

^1 ^Mean difference is calculated as control group value - intervention group value.

^2 ^independent T-Test, unadjusted

**Table 5:** ICCs and adjusted differences between groups

	ICC^1 ^district	ICC^1 ^surgery	adjusted^2^		
			
			mean difference	95%-CI	p-value
Primary outcome measure					

HbA1c (%)	-0.002	0.003	-0.03	[-0.15; 0.09]	0.607

Secondary outcome measures					

Creatinine (μmol/l)	0.001	-0.001	-0.96	[-4.16; 2.20]	0.545

Triglycerides (mmol/l)	-0.002	0.003	-0.10	[-0.24; 0.05]	0.190

Cholesterol (mmol/l)	-0.001	0.006	-0.10	[-0.21; -0.003]	0.043

HDL (mmol/l)	0.002	0.048	-0.01	[-0.05; 0.02]	0.438

LDL (mmol/l)	0.007	0.046	-0.02	[-0.10; 0.07]	0.684

RR systolic (mmHg)	0.002	0.054	-0.50	[-2.06; 1.05]	0.524

RR diastolic (mmHg)	0.009	0.045	-0.13	[-1.03; 0.76]	0.770

BMI	-0.001	0.020	-0.53	[-1.03; -0.02]	0.040

^1 ^calculated with GEE

^2 ^mixed models adjusted for baseline value and cluster structure

The number of days spent in the hospital was slightly lower in the intervention group (intervention 2.63 days, control 2.97 days, difference 0.34 days [95% CI -1.29; 0.61]), but the difference was not significant (p = 0.484 using mixed models).

Process quality measures including the percentage of patients receiving guideline-adherent foot-, eye-, and HbA1c-examinations show highly significant differences between the intervention and control group (table 6). Also, there were significantly more intervention patients (49.5%, n = 321) who participated in patient education than controls (20.1%, n = 169, p < 0.0001).

**Table 6:** Improvement of process quality by the DMP

Proportion of patients with	Intervention	Control	p-value^1^
eye examination	71.0%	51.2%	<0.0001

foot examination	73.8%	45.1%	<0.0001

patient education	49.5%	20.1%	<0.0001

regular HbA1c checks	44.1%	36.0%	0.002

^1 ^Fisher's Exact Test

## Discussion

This is the first study investigating the effects of a DMP designed and implemented by statutory public health insurance in a randomised controlled trial. Our data show that after one year, HbA1c was reduced in both the intervention and the control group favouring intervention. The small benefit, however, was lost after adjustment for baseline characteristics and ICC. Interestingly, ICC at the level of randomisation (districts) was negligibly small, but cluster effects at the surgery level were quite important. This finding suggests that DMP-effects and diabetes care are rather dependent on the motivation and effort of the participating physician than on the DMP. Only the positive effects of the DMP regarding weight reduction and lowering of cholesterol persisted after adjustment. Although these effects may very well be of clinical relevance, they are minor and need to be discussed in the light of the literature on the effectiveness of DMPs.

Four systematic reviews have been conducted on DMP's [21-24]. One showed that overall DMPs have positive pooled effect sizes regarding improvement in care [21]. However, effects seem to be largely dependent on the individual programme and its particular design. Thus, physician training improved guideline adherence in only 50% of the studies, improvement of disease control was reported in only 38%, and patient education was effective in only 44% of the trials. The review included experimental and quasi-experimental studies characterised by large heterogeneity and lack of quality. The most recent review presented similar results with only 24 out of 66 experimental or quasi-experimental trials on the effectiveness of DMPs showing significant improvement in patient care [22]. A systematic review performed specifically on programmes aimed at diabetes mellitus showed that DMPs may hold the potential to better long-term outcome due to improvements in glycemic control, i.e. a pooled estimate of HbA1c reduction by 0.5 percentage points [23]. However, the validity of this review is limited due to the inclusion of non-randomised studies and a significant heterogeneity amongst studies. The results of the fourth review are even less convincing due to the inclusion of all study types and settings (non-controlled observational studies as well as studies performed in HMOs [Health Maintenance Organisations] and Community Clinics) [24]. This review also reports a median HbA1c-reduction of 0.5 percentage points (range +0.2 to -5.9).

The HbA1c-reduction in pre-post analysis of our intervention group (-0.41 percentage points) corresponds to the reductions found in these reviews (0.5 percentage points). Our data suggest that the effect shown in these studies may largely be due to regression to the mean rather than to the effect of the DMP. Based on our data, the true effect is negligible if the HbA1c-decrease in the control group is taken into account and the result is adjusted for confounders. Thus our results confirm a known trend that effects from an intervention are usually overestimated in non-randomised studies.

DMPs can also be regarded as interventions that fit at least certain aspects of the chronic care model (CCM) as described by Bodenheimer et. al. [6]. Of the six components, the Austrian DMP contains three: the linkage to community resources (patient education classes), prioritising chronic care by the health care organisation (reimbursement of DMP), and self-management support (patient education and setting treatment goals by the patient). Delivery system design, decision support and clinical information systems have not been fully developed so far. By integrating these three pillars of chronic care into the DMP, the effectiveness could probably be strengthened. It has been shown in a meta-analysis that interventions for chronic diseases with at least one element of the CCM had beneficial effects on clinical outcomes and processes of care [7].

While the randomised design of our study is an important strength, some weaknesses must be considered. We need to take into account that the participation in a study itself may have an effect on physician performance and patient compliance [25]. As typical for pragmatic trials, blinding was not possible and the knowledge of being in the intervention or control group may have influenced the result. Nonetheless, the greater improvement in the intervention group regarding weight and cholesterol levels as well as the larger effects on process quality demonstrate that it may be worthwhile implementing DMPs and further develop the systematic disease management approach to improve chronic care, especially for patients who are motivated to participate in such programmes.

Even though we tried to achieve a study design free of bias, some risk of bias remains. First, only one third of the eligible Salzburg province physicians participated in the study which may favour selection of more motivated physicians who are early adopters. We could hypothesise that the effect of the DMP and especially physician education might have been larger if all physicians were included as less motivated physicians may reveal larger deficits in patient care thus giving a larger potential for improvement. Alternatively, the DMP would be of even less effect in a less motivated group of physicians, as suggested by the cluster effects at the physician-level in our study.

Second, patient selection may not have been free of bias as concealment of allocation was done at the physician level, but not the patient recruitment level. Differential patient recruitment was minimised by asking physicians to recruit consecutively and having control group patients sign up for DMP-participation after completion of the RCT. Baseline data similarity between the intervention and control groups shows that the recruitment method chosen largely resolved this problem.

Third, selection bias could have occurred due to physicians' selection of patients or due to a volunteer-based enrolment strategy in contrast to the "opt out" model used in the U.S. According to Linden et al., external validity may be compromised if programme participants do not adequately represent the population from which they were recruited [26]. Comparing our data with in-patient and out-patient data [5], we suspect that preferably "healthy" participants were recruited for our trial. However, this might also reflect healthier patient characteristics in primary care settings. No valid data representing patient characteristics in primary care are currently available in Austria. Thus, patients with higher HbA1c values may have been less motivated and less compliant despite their greater potential for improvement. Because this was a "pragmatic" study, a disproportionate recruitment of "healthy" patients may reflect real life, with recalcitrant cases not opting in for such programmes.

According to the extension of the CONSORT statement for pragmatic trials, special emphasis should be put on the generalisability (external validity) of the results [27]: Our trial is characterised by a high level of internal validity. Regarding external validity, the results can be transferred to a DMP implemented and carried out on a voluntary basis, but might not be completely applicable to mandatory participation of physicians and/or patients in a DMP. Also, the results of our study may not easily be transferable to the Austrian population as a whole. As stated above, diabetes prevalence in Salzburg is slightly lower than in the rest of the country, due to unknown reasons. The effect of the DMP on population health in our study may therefore be different and maybe less than the effect in a population with higher diabetes prevalence.

We cannot predict the influence of disease management on clinical outcomes based on our observation period of only 12 months. This problem has been addressed by other researchers when trying to determine long term benefits of DMPs [28,29]. The comparatively small benefit of the DMP on weight reduction, cholesterol level, and improvement of process quality is unlikely to result in significant changes of early clinical endpoints or all-cause mortality after only three years as has been hypothesised by Miksch et al. [9]. However, the United Kingdom Prospective Diabetes Study (UKPDS) demonstrated that even small differences in HbA1c may translate into benefits regarding acute and chronic diabetic complications as well as hospital admissions in the long run [30]. Therefore, a sufficiently powered, equally long follow-up-study will be necessary to confirm these effects for DMPs such as the Austrian "Therapie aktiv". The negative six-year-study from Denmark mentioned above does not have sufficient power to demonstrate any benefits regarding outcome or mortality [11]. We are planning to follow up both intervention and control patients enrolled in our trial for the following years to detect long term effects of the DMP.

## Conclusions

For the first time, the effects of a DMP implemented by statutory public health insurance have been evaluated in a randomised controlled trial. The Austrian DMP "Therapie aktiv" leads to significant weight and cholesterol reduction, and an improvement of process quality, but does not influence metabolic control as measured by HbA1c after one year. We conclude from our data that the effects of DMPs have probably been overestimated in earlier non-randomised trials, and that effect sizes in randomised trials may correspond to the strength of the intervention. The cluster effects at the level of the GP suggest that diabetes care depends more on the care offered by a specific GP than on the widespread implementation of a programme. Long-term studies are necessary to investigate whether the improvements in weight and cholesterol reduction as well as process quality translate into the prevention of diabetic complications.

## Competing interests

The authors declare that they have no competing interests.

## Authors' contributions

ACS: Conception and design of the study, data acquisition, analysis and interpretation of the data, drafting of the manuscript. HW: Conception and design of the study, data acquisition, analysis and interpretation of the data, critical revision of the manuscript. MF: Conception and design of the study, analysis and interpretation of the data, critical revision of the manuscript. SP: Statistical analysis and interpretation of the data, critical revision of the manuscript. PK: Data acquisition, interpretation of the data, critical revision of the manuscript. GK: Conception and design of the study, critical revision of the manuscript.

BF: Conception and design of the study, data acquisition, critical revision of the manuscript. RW: Conception and design of the study, critical revision of the manuscript. All authors read and approved the final manuscript.

## Authors' Information

ACS: MD, Professor of Family medicine, Director of the Institute of General Practice, Family Medicine and Preventive Medicine of PMU, specialist in chronic care and clinical studies

HW: Biologist, nutritionist, specialist in clinical studies

MF: MD, Assistant Director of the Institute of General Practice, Family Medicine and Preventive Medicine of PMU, specialist in public health

SP: Statistician at the Institute of General Practice, Family Medicine and Preventive Medicine of PMU

PK: MD, GP, Director of the Workinggroup for Preventive Medicine, Salzburg (AVOS)

GK: MD, Chief Physician of the Styrian Public Health Insurance, Graz, Austria

BF: MD, GP, former Vicepresident of the Austrian Association of General Practice (ÖGAM)

RW: MD, Associate Professor, Director of the Medical Department of the Diakonissen-Hospital, Salzburg

## Pre-publication history

The pre-publication history for this paper can be accessed here:

http://www.biomedcentral.com/1471-2296/11/86/prepub
